# Unmasking the Perky Effect: Spatial Extent of Image Interference on Visual Acuity

**DOI:** 10.3389/fpsyg.2012.00296

**Published:** 2012-08-15

**Authors:** Adam Reeves, Catherine Craver-Lemley

**Affiliations:** ^1^Department of Psychology, Northeastern UniversityBoston, MA, USA; ^2^Department of Psychology, Elizabethtown CollegeElizabethtown, PA, USA

**Keywords:** visual imagery, Perky effect, spatial vision, unmasking

## Abstract

We have previously argued that visual mental images are not substitutable for visual percepts, because the interfering effects of visual stimuli such as line maskers on visual targets differ markedly in their properties from the interfering effects of visual images (the “Perky effect”). Imagery interference occurs over a much wider temporal and spatial extent than masking, and unlike masking, image interference is insensitive to relative orientation. The lack of substitutability is theoretically interesting because the Perky effect can be compared meaningfully to real line masking in that both types of interference are visual, not due to optical factors (accommodative blur or poor fixation) or to high-level factors (attentional distraction, demand characteristics, or effects of uncertainty). In this report, however, we question our earlier position that spatial extents of interference are markedly different: when images and real lines are matched in contrast, which was not done previously, their interference effects have very similar spatial extents. These data add weight to the view that spatial properties of images and percepts are similar in respect to extent. Along with the wider temporal extent and the insensitivity to orientation, the new results remain compatible with our older hypothesis that to create a clear mental image in a region of visual space, incoming signals from the eye must be suppressed (Craver-Lemley and Reeves, 1992). We have pursued this idea in this report using “unmasking,” in which adding elements to the visual image in the region beyond the zone of suppression reduces the Perky effect.

## Introduction

Whether or not visual mental images act like visual percepts is a fascinating and important question in cognitive science. Should they have the same functions and share the same anatomy, as has been argued from imaging studies of cortical area V1, one might expect them to be mutually substitutable or at least, analogous to each other. That images and percepts both have spatial layout, both contain rich visual information, and both share the functional property of reduced acuity in the periphery (Kosslyn, 1980), may be taken to argue in favor of substitutability; in Kosslyn’s array theory, for example, images and percepts are thought to share the same representational array, and in Finke’s (1980) theory, images and percepts are functionally equivalent for high imagers. In our earlier work, we argued in favor of analogous spatial behavior but against substitutability (Arterberry et al., 2002), based on empirical findings concerning the so-called Perky effect, in which visual images, analogous to real masking stimuli, can suppress perception of real visual targets (as discovered by Perky, 1910). The Perky effect is ubiquitous in studies of percept-image interactions, with the imagined stimulus depressing sensitivity to briefly flashed visual targets by 15% or better (0.8 d′ units or more; Segal and Fusella, 1970; Craver-Lemley and Reeves, 1987, 1992; Ishai and Sagi, 1997). In the Perky experiments, the experimenter requested that the subject project his or her visual image in the location of the visual target. Thus when the target was flashed, it was presumably presented at the same location in visual space that the image already occupied (the image-on-target, or “ON” condition). Such images always interfere with perception of the target by lowering sensitivity, not by making the response criterion sub-optimal. Interference occurs in the brain, not the eye, as controls have ruled out optical effects such as poorer accommodation of the lens during imagery or poorer fixation during imagery (Craver-Lemley and Reeves, 1987). Moreover, the Perky effect does not stem from demand characteristics; participants told that imagery aided perception produced as great a Perky effect as those told it would hinder perception (Craver-Lemley and Reeves, 1987, Experiments 1a and 1b). Other experiments have ruled out mere distraction, the notion that having any image would add a load to the cognitive system and thus lower visual performance, as auditory images have virtually no effect on visual target (Craver-Lemley and Reeves, 1992; although they do on auditory ones: Segal and Fusella, 1970), visual images located far away from the visual target have almost no effect (Craver-Lemley and Reeves, 1987; Craver-Lemley and Arterberry, 2001), and diverting attention to a distracting light does not increase the interference (Craver-Lemley and Reeves, 1992). Thus we have used the Perky effect as a test-bed for discovering whether images and real visual stimuli have the same functional properties. We adopted an acuity target as the visual stimulus, since the visual properties of acuity stimuli are well documented^1^.

Experiments using real (rather than imagined) stimuli have revealed several well-known characteristics of masking of line targets, including that masking is reduced rapidly as the masking stimuli are moved away laterally from the target; that masking is reduced if the orientation of the mask differs from that of the target, disappearing if they are at right-angles to each other (although this general rule may not be true above threshold for dichoptic low-spatial frequency gratings: Meese and Hess, 2004); that masking is eliminated if the target-to-mask temporal asynchrony is more than 200 ms; and that patterned masking is cortical – unlike noise masks, patterned masks are as effective when the target is presented to the opposite as to the same eye (see, e.g., Westheimer, 1965; Westheimer and Hauske, 1975). Relying on this literature, we argued that interference due to images (the Perky effect) had quite different characteristics from real masking (Craver-Lemley and Reeves, 1992), in that the Perky effect is independent of the relative orientation of the image and the real lines, and the Perky effect persists up to 6 s after the image has been removed (Craver-Lemley and Reeves, 1987). Moreover, the Perky effect apparently covers a wider region of visual space than does the interfering effects of real lines (Craver-Lemley and Reeves, 1992).

In this past research, we asked participants to create visual images but we have not matched the contrasts of the mental images to the contrast of the physical stimuli. Typically, we employed high contrast physical stimuli, whereas participants created mental images *ad lib*, and our participants had informally reported that their imagined lines were lighter and less distinct than those pictured (Craver-Lemley and Reeves, 1987, 1992). We therefore wondered if our complete rejection of substitutability was too hasty, the difference being, not between real and imaged stimuli, but between high and low contrasts. We did not repeat our earlier work on image orientation because it is already established that masking by low-contrast gratings, like masking by high contrast ones, disappears at the orthogonal orientation, implying that our earlier result (orthogonal images interfered as much as parallel ones) would differ from real line masking no matter what real line contrast was used. We also did not repeat the work comparing timing, as the Perky effect lasts for 4–6 s after an image has been terminated, long after any masking by real lines is complete. However, we were concerned to repeat our work on the lateral extent of interference. We therefore compared the effects of images of lines against not only the real black lines used before, but also against real gray lines matched in contrast with the participant’s own imagined lines (Experiment 1). We continued to use lines rather than other acuity targets for continuity with our previous work; however, we note here that the spatial structure of the stimulus may affect the lateral extent of interference. While Ishai and Sagi (1997), like us, found considerable interference by imagery in the “ON” condition when using Gabor (wavelet) targets, they also found a small but significant *enhancement* in sensitivity when the mental image was displaced just one wavelength away from the target center. With lines, interference is also reduced by increasing target-image distance, but we have not found enhancement.

## Experiment 1

This experiment was conducted to find out whether gray lines that were matched in contrast to images of lines would interfere with acuity in the same manner as the mental images. Black real lines were also used to replicate previous work. Target-image and target-real line distances were varied to permit comparison of the lateral extents of image interference and real line masking.

## Materials and Methods

### Participants

Eleven undergraduate volunteers enrolled in an introductory psychology course participated in this study for payment. All participants had normal or corrected-to-normal vision and had no previous experience in vision or imagery experiments.

### Apparatus and stimuli

Experiments were conducted with Model T-2B-1 two-field Gerbrands tachistoscopes. The fixation and test fields were 58 cm from the eyes, superimposed by a half-silvered mirror, and surrounded by complete darkness (the inner walls of the apparatus were covered in black velvet). The *fixation* field was a white 17 cd/m^2^ rectangular index card that subtended 10° × 15° at the eye. Central fixation was aided by two small (1.22 mm) black dots placed 5.2° apart, symmetrically above and below the center of this field. Small guide dots (Experiments 1 and 2) or guide lines (Experiment 3) were added at top and bottom of the fixation field to aid the subjects in locating their images when images were requested.

There were two real line conditions, in which imaginary lines were not requested, namely, Black Lines and Matched Gray Lines. In these conditions, 1.0 cm wide and 7.6 cm high black or gray lines, subtending 0.1 × 7.5°, were added to the fixation field. Being part of the fixation field, these lines were presented continuously, unlike the vast majority of studies of masking in which masking lines are flashed; however, continuous presentation was necessary to mimic the imaginary lines, which were imagined throughout each trial, as closely as possible.

The *test* field was used to flash the vernier acuity targets, as in Craver-Lemley and Reeves (1992); Craver-Lemley et al. (1997). The test field was dimmed to 6 cd/m^2^ so that flash duration at threshold would not be lower than 10 ms for the best participants. Each target was made of two, thin black (75% contrast) lines, each line subtending 2.2° vertically and 0.1° horizontally, with a vertical gap of 0.5° between them. As the vertical gap between the acuity targets is relatively large, hyperacuity was not achieved. The targets (black tape by Chart-Pak) were mounted on white index cards. The lower of the two lines in each target was, with equal probability, offset to the left or right of the upper line. There were 10 different offsets ranging from 4.2 to 22 min visual angle, with a mean offset of 9.7 min. The target presentation was randomized. The participant’s task was to report whether the lower line was to the right or left of the upper line; they were told that each was equally likely to occur. The test field was jittered randomly left or right by up to 17 arc min from trial to trial, to encourage the participant to judge the lines relative to each other rather than relative to the fixation point.

#### Conditions

There were 10 conditions, run in separate blocks:

##### Imaged lines

**Figure 1:**
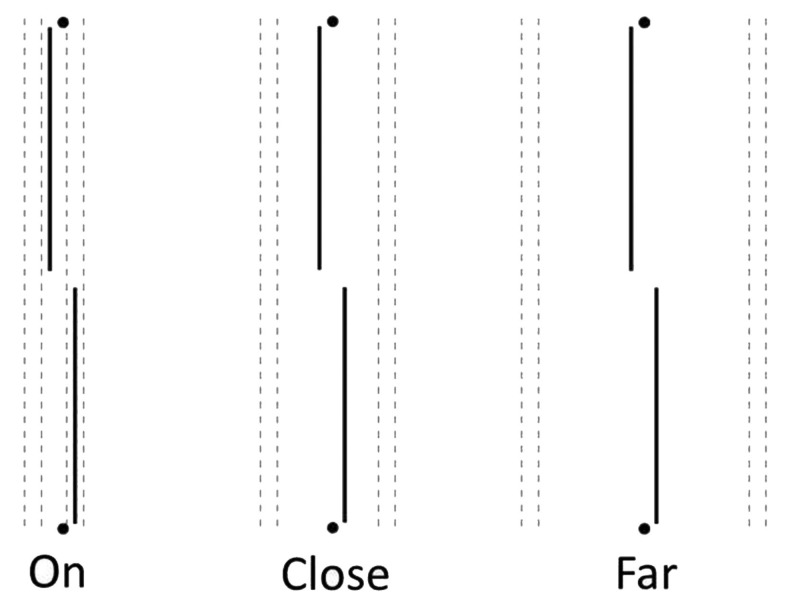
**Two-line acuity targets surrounded by imaginary lines, ON, CLOSE, or FAR away, as directed by small guide spots or lines at top and bottom of the fixation field (not shown)**. Real black or gray lines were positioned in the same places as the imagined ones.

During imagery trials, participants were asked to evoke a mental image of four black vertical lines. Participants were shown a picture of the lines they were to imagine and asked to locate their image in reference to small guide marks penciled in at the top and bottom of the fixation field. Each participant’s acuity was measured while they were instructed to project mentally vertical line images either “ON” the acuity target (nearest line 0.1 cm, or 6 min arc, away from the target), “CLOSE” to the target (nearest line 0.8 cm, or 0.8°, away), or “FAR” from it (innermost line 2.0 cm, or 2.0°, away; see Figure 1). Guide marks indicated to the participants where to place their mental images. They were given ample time to comply, and none reported difficulty in locating their imaginary lines with respect to the guide marks. They were instructed to hold their images throughout the trial; if reported losing the image, the trial was re-run.

##### Black lines

During black line trials, the four real, thin black lines were shown continuously from top to bottom of the fixation field. These lines appeared either “ON” the acuity target, “CLOSE” to it, or “FAR” from it, with the same spacing as for the imaged lines.

##### Matched gray lines

During the initial practice session, we asked each participant to select lines from a prepared set (ranging in color from light gray to black) which best matched his or her imagined lines. All participants selected real gray lines that were lighter than the black lines, matching images to Munsell color chips between 10B 8/1 and N8/5. As in the Imagery and Black Lines conditions, the Matched Gray Lines (at the level selected by each participant) were presented in the same ON, CLOSE, and FAR conditions.

##### No imagery

In the baseline condition, accuracy was measured for reporting the acuity target, without either real or imagined lines.

Thus the 10 conditions comprised the baseline with no lines and no imagery, three conditions with imagined lines (ON, CLOSE, FAR), three with Black Lines (ON, CLOSE, FAR), and three with Matched Gray Lines (again, ON, CLOSE, FAR).

#### Procedure

Participants were individually tested during two 1 h sessions. The duration of target presentation was determined during an additional 15 min practice session. Participants were told to fixate between two fixation points where the target would be presented. The target duration was adjusted until accuracy in the baseline no imagery acuity task was close to 90%. Target duration remained fixed after the practice session; it averaged a nominal 8 ms (range: 3–25 ms; as these values are well below the critical duration, target duration exerts its effect via energy). Participants reported the direction of offset (left, right) on each trial.

Conditions were run in separate blocks of 25 trials in each of two sessions. The order of the blocks was randomized over participants. There were 25 trials per participant in the black lines ON condition, which, being near chance in the first session was not re-run, however 50 trials were included for each of the other nine conditions.

Instructions were reviewed before each block. In the No Image and the two real line conditions, the experimenter initiated each trial by saying, “go” and then the acuity target was presented. In the imagery condition, participants indicated their image was ready by saying “go,” and the experimenter then presented the acuity target. Participants were requested to re-create or re-evoke their images on each of the 25 imagery trials during a block. Participants were allowed to rest between blocks. They did not receive any feedback until after the second session had been completed.

Our experimenters were undergraduate research assistants who were naïve as to the predicted outcomes of the experiments and had never before collected data in imagery experiments. They could not see the acuity target until after each trial had been completed, and so were unaware of what the correct response should be prior to stimulus presentation and the participant’s response. Individual data were not tabulated or analyzed until after the experiment was completed.

## Results and Discussion

**Figure 2:**
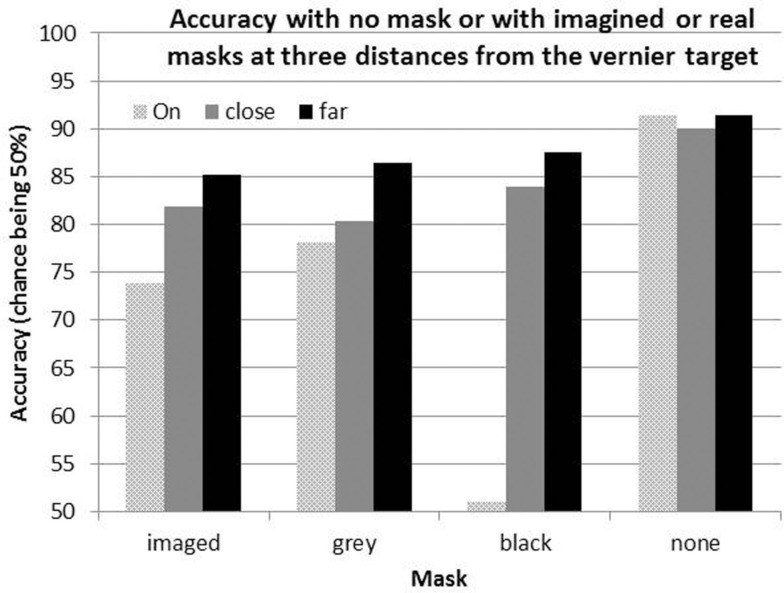
**Mean correct performance for the acuity task with imaged lines, with real gray lines matched to the participant’s images, with real black lines, and in the baseline no image, no mask (“none”) condition**. The interfering lines are ON the acuity target, CLOSE to it, or FAR from it.

Accuracy in the baseline (No Image) condition averaged 91.4% correct across participants, reflecting successful choices of target durations during initial practice. Accuracy in all other conditions was lower than this, reflecting the interfering effects of real and of imagined lines. An omnibus analysis of variance (ANOVA) revealed a main effect of both distance [*F*(2,9) = 191.54, *p* < 0.001, $\eta_{\text{p}}^{\text{2}}\text{ = 0}\text{.95}$] and line type [*F*(2,9) = 23.03, *p* < 0.001, $\eta_{\text{p}}^{\text{2}}\text{ = 0}\text{.70}$], as well as an interaction [*F*(4,7) = 45.66, *p* < 0.001, $\eta_{\text{p}}^{\text{2}}\text{ = 0}\text{.82}$]. Mean percent correct in each of the conditions is plotted in Figure 2,^2^. As expected, the results show a clear Perky effect, as imagined lines reduced acuity. The reduction is 17.6% when the imagined lines are “ON” the target, in line with Segal and Fusella (1970), Reeves (1980), and Craver-Lemley and Reeves (1987, 1992). The Perky effect was attenuated if the image was shifted off target, in the present research from 17.6 to 6.3% (i.e., baseline No Image – image OFF), also in line with Craver-Lemley and Reeves (1987).

The critical new result is that (real) Matched Gray lines have rather similar effects on acuity as imagined lines, reducing performance by 13.2% in the ON condition and 5% in the FAR condition, compared to 17.6–6.3%, respectively. Given the difficulties inherent in exactly matching the contrast and spatial position of the real lines, one is struck by the congruence between these effects and those for imagined lines. This result causes us to re-think our original claim that the spatial extent of interference by images exceeds that of real lines; contrast, not reality, appears to be the critical variable, and when contrasts are matched, the reductions in interference with distance from the target are similar.

## Experiment 2

**Figure 3:**
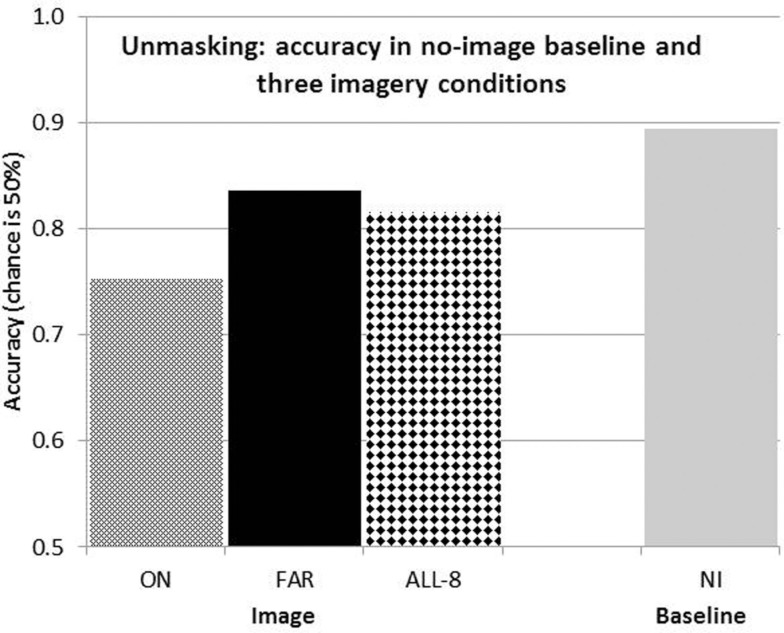
**Mean correct performance for the acuity task with ON and FAR images, each of four lines, with ALL eight imagined lines, and in the baseline no image condition**.

Experiment 1 suggests that contrast plays a major role in the Perky effect, but does contrast polarity also matter? We had not compared images with positive and negative contrasts in previous studies of the Perky effect, nor have we located a study that has. We therefore asked participants to create images of white vertical lines for comparison with the images of black vertical lines used before, projecting both onto a gray field. With real stimuli, acuity depends strongly on the absolute value of the contrast, but when targets are presented on a constant background, acuity is only weakly dependent on contrast polarity (Pointer, 2001), as is metacontrast (Breitmeyer et al., 2008: note the same polarity masks and targets in his Figure 3) and object substitution masking (Backmann and Luiga, 2008). (We note that earlier studies of polarity compared acuity for white targets on a black background with acuity for black targets on a white background, but these backgrounds alter adaptation level, light scatter, and pupil size; a constant background controls these factors.) If the analogy between imagery and real lines holds true of contrast, we therefore expect that contrast polarity of the image would leave the magnitude of the Perky effect relatively unchanged, given that we use a constant lit background.

## Materials and Methods

### Participants

Twenty-two undergraduates with normal or corrected-to-normal vision volunteered to participate in exchange for course credit in an introductory psychology course. None of the students had previously participated in an imagery experiment before.

### Materials and procedure

The stimuli and apparatus were as in Experiment 1. Participants were again tested individually in single 45 min. sessions. The acuity targets were presented for a duration that resulted in close to 90% correct performance without imagery, chosen for each participant during an initial 15 min practice session. The mean nominal target duration was 9.3 ms (range = 3–30 ms). The task was, as before, to report whether the lower line was offset to the left or right of the upper line.

There were three conditions: No Image (the baseline), Imagine Black Lines, and Imagine White Lines, ON the target. The three conditions were not blocked but were randomly intermixed. There were 50 trials per condition for a total of 150 trials for each participant. Participants were cued prior to the beginning of each trial as to whether they were to imagine four vertical white lines, four vertical black lines, or to have no image at all. During imagery trials, participants indicated when they were ready with the image, and then the experimenter presented the target. Participants were instructed to maintain the image for the duration of the trial. On No Image trials, the experimenter indicated it was a no image trial and then said, “go” to alert the participant that the trial was beginning. Participants received a 5 min break after 75 trials.

## Results and Discussion

Correct performance, averaged over targets and participants was 89.1% with no image, 80.8% for Imagine Black Lines, and 79.0% for Imagine White Lines. A significant condition effect, *F*(2,42) = 23.71, *p *< 0.001, was found in a one-way repeated measures ANOVA, with both Perky effects significant by Tukey’s honestly significant difference test (*p *< 0.01). However, accuracy with Black line images (80.8%) did not differ from that with White line images (79.0%).

The Perky effect for Imagine Black Lines, being 8.3%, was smaller than the 17.6% effect found for ON images in Experiment 1. Since otherwise these conditions were the same, this difference in magnitude may be due to randomizing rather than blocking the conditions. Randomizing may reduce magnitude because the Perky effect lasts several seconds after the participant reports turning off the image (Craver-Lemley and Reeves, 1987), and therefore some image-generated interference may leak into those baseline trials which directly follow imagery trials. However, randomizing has the advantage that the lack of a difference between black images and white images is unlikely to be due to a change in strategy between conditions. We conclude that the lack of a difference is genuine, and therefore that contrast, but not contrast polarity, influences the Perky effect. This finding for imagery appears to agree with the literature concerning the effects of contrast polarity on acuity for real stimuli, but this agreement is not definitive as we have not studied the effects of contrast polarity with real lines in our equipment and our target-line distances.

## Experiment 3

If the analogy between real and imagined lines does hold up, at least for extent if not for orientation, then it is possible that imagined lines, like real ones, might show “unmasking” (also termed “release from masking.”) In this phenomenon, a real mask superimposed on the target has its masking effect reduced by adding a distant mask, even when the latter had little direct effect on target visibility (Haber, 1970). It is as if the distant mask releases the target from masking, either by inhibiting the superimposed mask (Dember and Purcell, 1967), or by strengthening the target (Briscoe et al., 1983). Herzog (2007) reviews unmasking in a more complex spatial arrangement when the vernier is masked by a spatial grating. We wondered whether the same phenomenon of unmasking could be found with visual images.

## Materials and Methods

### Participants

Sixteen undergraduates with normal or corrected-to-normal vision volunteered to participate in exchange for course credit in an introductory psychology course. None of the students had previously participated in an imagery experiment before.

### Materials

A Gebrands two-field tachistoscope was used, of the same type as the one used in Experiments 1 and 2. Stimuli were the same as before. On imagery trials, participants were asked to evoke four or eight black vertical line images. Small guide lines in the fixation field helped the participants locate their images. Participants imagined four vertical lines in the ON and FAR conditions illustrated in Figure 1, but in the *new* condition, ALL-8, they were requested to generate an image of eight black lines, as indicated by the guide lines. Ideally this new image is the sum of ON and FAR images.

### Procedure

As before, participants were tested individually in a single session lasting approximately 50 min. The acuity targets were presented for a duration that resulted in close to 90% correct performance in the baseline (no imagery) condition, which was chosen for each participant during an initial 15 min practice session and thereafter fixed. The mean duration of targets was 13 ms (range = 4–33 ms). All three imagery conditions were practiced until the participant was familiar with the procedure. Participants were asked to maintain central fixation during the experiment. The task was, as in Experiments 1 and 2, to report whether the lower line was offset to the left or right of the upper line. The three imagery and one no imagery conditions were randomly intermixed with 50 trials per condition for each participant. Participants were cued prior to the beginning of each trial as to what to image, or to have no image at all. During imagery trials, participants indicated when they were ready with the image and then the experimenter presented the target. Participants were instructed to maintain the image for the duration of the trial. On baseline trials, the experimenter indicated it was a no image trial and then said, “go” to alert the participant that the trial was beginning. Participants received 5 min breaks after each block of 50 trials.

## Results

The differences between the four conditions were highly significant [*F*(3,45) = 9.48, *p* < 0.01] by a repeated measures, one-way ANOVA applied to the scores of the 16 participants. Mean accuracy was 89.4% in the baseline “No imagery” condition, reflecting successful manipulation of the individual target durations. Accuracy with ON images was 75.3%, giving a reduction in accuracy, or Perky effect, of 14.2% (Figure 3). When the lines were imaged far from the target, accuracy was 83.6%, for a much smaller Perky effect of 5.8%. These two Perky effect magnitudes are similar to those reported before by Craver-Lemley and Reeves (1987) for these two conditions. They suggested to us that the Perky effect is spatially localized. The new condition is ALL-8, in which both ON and FAR lines were imaged; in this case, mean accuracy was 81.6%, for a Perky effect of 7.8%.

The Perky effect in ALL-8 would be greater than in ON if the effect of the inner lines summed with that of the outer lines, because the inner lines were the same in ON as in ALL-8, and the outer lines had a weak (7.6%) effect of their own. However, the results showed a clear diminution of the Perky effect in ALL-8, to a mere 7.8%, which is much less than additive.

## Discussion

There are three possible explanations for our new result, that the Perky effect with eight imaged lines is less than additive: suppression, distraction, and weakening. We will ultimately argue for an explanation in terms of suppression, but the other two possibilities merit consideration, especially as the result from Experiment 3 is singular and we therefore do not have an adequate sampling of experimental conditions to permit a definitive conclusion.

By “weakening” we mean that relative to the strength of the ON image, that of the ALL-8 image is reduced, either by graying, blurring, making less vivid, decreasing image duration, or otherwise lowering image quality. The ALL-8 image could in principle be weaker because maintaining eight vertical line images is simply more taxing than maintaining four. However, both in initial practice and in debriefing, we found no evidence for this: all of the participants reported being able to imagine eight lines as well as four. In debriefing, we again asked whether the three images (ON, FAR, and ALL-8) had equal vividness, and had kept their vividness throughout the experiment; participants reported that they had. Such retrospective reports do not disprove the possibility of some weakening, but as explicitly weakening the ON image by asking participants to gray-out their imagery reduced the Perky effect by only 4%, as we found in a pilot experiment, we think the consequences of any slight weakening would be marginal.

By “distraction” we mean that having a vertical line image might distract attention from the primary task of reporting the acuity target. We originally argued that images required the same amount of attention, and were equally distracting, whether they were near the acuity target or far from it, so differences between them in the magnitude of the Perky effect could not be explained by attention (Craver-Lemley and Reeves, 1987, 1992). One might dispute this logic, but note that ON images demand that attention be paid to the area in which the target will be presented, as opposed to FAR images which draw attention away, so distraction would predict more, not less, of a Perky effect in FAR than in ON. Moreover, Craver-Lemley and Reeves (1992) explicitly manipulated attention by having participants attend to a blinking light in the periphery; this lowered accuracy overall but left the Perky effect unaltered. Again, Craver-Lemley and Reeves (1987) found that a vertical line image produced a Perky effect for 4–6 s after the subject indicated that he or she had removed the image, and in this situation there was no image to distract attention. Finally, Craver-Lemley et al. (1997) found that having vertical line images in front of the visual target produced a Perky effect, but having them behind the target had no interfering effect at all. Since to control location in depth, the same image was requested on one or other face of an outlined cube, it is unlikely that differential attention to the image could explain this result. We regard all this as good evidence that the Perky effect is not entirely due to distraction of attention.

By “suppression,” we invoke a hypothetical mechanism in which the inner four lines in ALL-8 have their interfering effects on the target reduced by the outer four lines. Although it is somewhat counterintuitive that adding masking elements could reduce masking, this effect is known in the masking of visual targets by real maskers (Dember and Purcell, 1967; Herzog, 2007), and the same idea has been applied before to the unmasking of real stimuli by visual images (Reeves, 1980). Either the outer lines “unmask” the target by suppressing the interfering effect of the inner lines, or the outer lines act to facilitate the target. Facilitation by distant imagery is theoretically possible and has been observed using Gabor-like stimuli and images by Ishai and Sagi (1997); however, their effect, thought theoretically important, was relatively small. We are inclined to conclude that unmasking occurs when the active part of the mask is itself masked due to some form of inhibition by additional mask elements, both for real lines and for imaged ones. However, the idea that the far away image elements inhibit the ones close to or on the acuity target is based on this single result, and this may not survive further testing; and even if it does, the properties of image unmasking may turn out quite different from those of real line unmasking.

## General Discussion

Craver-Lemley and Reeves (1992) suggested that the Perky effect is a consequence, not of the image directly, but of a mechanism designed to *protect the image* by inhibiting visual input from the region of the visual field in which the subject was requested to have the image. Since we found the Perky effect was insensitive to the relative orientation of imaged lines and real line acuity targets, we suggested that the protection was provided by inhibitory cortical feedback to the LGN, the last relay in the visual pathway which is not sensitive to orientation. Our measurements suggested that the feedback effect was weak, amounting to a reduction in stimulus contrast of 0.24 log units, but this was enough to account for the reduction in acuity that we had reported. As already remarked, we have rejected other explanations of interference, such as poorer optical accommodation, shifts of gaze away from the target, confusion of the target with the image, distraction of attention, response bias, and experimenter effects, all based on empirical data.

However, the notion of a protective mechanism operating at the level of the LGN did not predict the finding of Craver-Lemley et al. (1997) that having vertical line images behind the visual target eliminates the Perky effect, because LGN neurons are insensitive to depth as well as to orientation. The model could be modified such that the feedback signal itself is dependent on depth, such that only frontal images need protection from visual input, although this is quite *ad hoc*. However, the new result, of unmasking, cannot be explained in such a manner. An alternative hypothesis suggested by Perky (1910) and by Segal (1971), and resurrected by Craver-Lemley et al. (1997) in order to explain the effect of depth, is that the Perky effect results from a combination of real and imagined features that makes the real features more difficult to extract. This notion is attractive in many ways, but it presupposes that imagined features have much more featural spread than real ones, since, unlike real lines, horizontal line images interfere as much with acuity for vertical lines as do vertical line images. It also supposes that some unknown after-effects of imaged lines, not just concurrent images, combine with stimulus features, as images continue to interfere for 4–6 s after being removed (Craver-Lemley and Reeves, 1987). It may be that some combination of the two hypotheses, or perhaps an altogether different one, will eventually explain the intricacies of the Perky effect.

## Conflict of Interest Statement

The authors declare that the research was conducted in the absence of any commercial or financial relationships that could be construed as a potential conflict of interest.

## Footnotes

^1^Recent research has re-emphasized Hume’s view that the vividness of mental imagery is functional. David Hume had argued that images were less vivid than the reality they represented, and that such a difference was sufficient to distinguish truth from fantasy. Perky (1910), however, showed the participants do not always reliably distinguish images from percepts, and perhaps more tellingly, reported image vividness does not in general correlate with perceptual ability though it may with memory (Hilgard, 1981). Indeed, we (Craver-Lemley and Reeves, 1987) found no correlation between the self-reported vividness of visual imagery and the extent of the Perky effect across 125 participants. However, Rodway et al. (2006) recently reported that while self-reported high and low vividness participants did not differ in overall levels of change detection accuracy, high vividness participants were more accurate at detecting salient changes to pictures. Moreover, self-reports of image vividness correlate *r* = 0.73 with image strength as documented by the fMRI (Cui et al., 2007), and stronger visual images are more likely to survive size-scaling than are weaker ones (D’Angiulli and Reeves, 2002). The strength of simple line images, as manipulated by the participants themselves, therefore seemed like a possible factor in our experiments.

^2^A detailed analysis of the data of Experiment 1 reveals the effects expected from eyeballing Figure 2. In the ON conditions, black lines showed significantly more interference than both matched lines [*t*(10) = 7.41, *p* < 0.001], and imagined lines [*t*(10) = 5.71, *p* < 0.001], and imagined lines showed significantly more interference than matched lines [*t*(10) = −6.71, *p* < 0.001]. In the CLOSE conditions, matched lines showed significantly more interference than black lines [*t*(10) = −2.86, *p* = 0.017], imagined lines showed significantly more interference than black lines [*t*(10) = −3.32, *p* = 0.008], and matched lines and imagined lines were not significantly different, *t*(10) = 1.44, *p* = 0.181. In the FAR conditions, black lines and matched lines were not significantly different [*t*(10) = −1.35, *p* = 0.208], but imagined lines showed significantly more interference than black lines [*t*(10) = −2.71, *p* = 0.022] and matched lines [*t*(10) = −2.28, *p* = 0.046]. Images ON the target showed more interference than both images CLOSE to it [*t*(10) = 4.58, *p* = 0.001] and FAR from it [*t*(10) = 5.14, *p* < 0.001]; also, CLOSE images showed more interference than FAR images [*t*(10) = 4.50, *p* = 0.001]. Matched Gray Lines ON and CLOSE to the target did not differ [*t*(10) = 1.36, *p* = 0.204], but they interfered more than those FAR from it [*t*(10) = 3.9, *p* = 0.003, and *t*(10) = 4.54, *p* = 0.001]. Real Black Lines ON the target interfered more than both those CLOSE to it [*t*(10) = 11.73, *p* < 0.001] and FAR from it [*t*(10) = 14.66, *p* < 0.001], and those CLOSE interfered more than those FAR [*t*(10) = 7.61, *p* < 0.001]
